# Assessing the Impact of Social Media Use on Everyday Emotion in Health Crises: A Study of International Students in China during COVID-19

**DOI:** 10.3390/healthcare9081011

**Published:** 2021-08-06

**Authors:** Ebenezer Larnyo, Baozhen Dai, Jonathan Aseye Nutakor, Sabina Ampon-Wireko, Ruth Appiah, Abigail Larnyo, Benedicta Akey-Torku, Edmund Nana Kwame Nkrumah

**Affiliations:** 1 Department of Health Policy and Management, School of Management, Jiangsu University, 301 Xuefu Road, Zhenjiang 212013, China; dr.ebenlarnyo@ujs.edu.cn (E.L.); jnutakor@gmail.com (J.A.N.); amponwirekosabina@gmail.com (S.A.-W.); ruthapp27@yahoo.com (R.A.); benedicta_akey@yahoo.com (B.A.-T.); Nkrunak@gmail.com (E.N.K.N.); 2 College of Basic and Applied Sciences, University of Ghana-Legon, Accra P.O. Box LG 25, Ghana; akusiator@wacci.ug.edu.gh

**Keywords:** adult depression, generalized anxiety, everyday emotion, social media, international students, COVID-19

## Abstract

Social media has become a valuable tool in providing an opportunity to stay in touch with one’s social networks, providing reassurance and practical advice to individuals to pre-empt panic and rumors in COVID-19. However, the implications of social media use on the everyday emotion (anxiety and depression) of users especially, international students, are not well understood. Thus, this study sought to examine the impact of social media use on the everyday emotion of international students in China during COVID-19. Using a structured online questionnaire based on modified questions from the generalized anxiety disorder 7 (GAD-7) and the Patient Health Questionnaire 9 (PHQ-9) and social media use instruments, data were collected from 480 participants. Of the total responses received, 474 were further analyzed employing the Partial Least Squares Path Modelling (PLS-PM). This study showed a significant positive relationship between social media use and everyday emotion (B = 0.34, 95% CI (0.26, 0.44)). Additionally, self-rated anxiety and depression associated with social media use among international students were generally mild (*n* = 249, 52.50% and *n* = 350, 73.80%, respectively). Moderating effects revealed that age and sex do not significantly moderate the relationship between social media use and everyday emotion in COVID-19. Given the nature of social media among international students, who are also prone to suffering from anxiety and depression associated with social media use, the positive effect of social media use and everyday emotion, especially in COVID-19, has important implications for international students’ education stakeholders. Thus, gaining a deeper understanding of this relationship could enable them to harness social media and use it as a valuable tool to overcome the social distancing constraints in COVID-19.

## 1. Introduction

Since its emergence, social media has been instrumental in the everyday activities of individuals in various aspects of their lives, including personal communication, business development management, and networking.

In risk communication during health crises, the use of social media as a source of delivering and receiving health information alerts, conveying public opinions and attitudes on issues related to infectious diseases, public health, and behavioral medicine has, over the past decade, expanded tremendously [1,2,3]. Since the outbreak of COVID-19 in December 2019 [4], public health officials, institutions, and other governmental organizations have instituted widespread lockdowns and other rigorous and stringent measures to curtail the spread of the virus among their populations. These measures were targeted at discouraging large gatherings and encouraging citizens to stay at home as much as possible, eliminating an essential stress management tool: social support systems. Consequently, this situation has resulted in many people turning to and spending extra time on social media to maintain some semblance of social network systems and search for relevant information concerning the COVID-19 pandemic [5].

Among the popular groups of people familiar with social media is the youth, specifically, students studying abroad. Undoubtedly, international students use social media to socialize, educate, and constantly communicate with their families and friends [6]. Previous studies have further shown that the use of social media reduces barriers to group interactions and telecommunications among students [7,8], supports collaborative learning activities [9], encourages self-learning [10], and fosters students’ engagement and motivation [11]. In this era of the COVID-19 pandemic, the use of social media has particularly become very valuable in the lives of international students due to the several lockdown restrictions imposed by most school authorities in the bid to curtail the spread of the virus. Thus, most international students had no other option than to resort to social media, using it as a tool to stay connected with their families and social networks in their home countries and also access COVID-19-related information. This tends to make them heavier users of social media, the Internet, and other digital communication tools compared with the general population [12] and social media users in non-pandemic situations. In China, social media use has been found to mitigate the impact of mental health problems during COVID-19 and lockdowns by serving as a tool for maintaining social support during physical distancing and providing health information, telemedicine, and online psychological counseling [13,14].

Regardless of the enormous benefit derived from the use of social media, especially among international students in the hit of the pandemic, some studies have shown that spending extra hours on social media may pose behavioral threats of addiction, depression, and anxiety [15,16,17]. Studies have also shown the manifestation of mood swings, cognitive decline, adverse physical and emotional reactions, and interpersonal and psychological issues, to be consistent with symptoms of social media addiction [18,19]. One cannot underestimate that a global pandemic of such magnitude may trigger depression and anxiety among individuals [20]. This experience may even be worst among international students studying in a new environment miles away from home.

The study of Bontcheva et al., 2013, and Roth and Brönnimann, 2013, opined that, due to the incidence of information overload or misinformation in the use of social media, users might tend to have increased mental health problems [21,22]. Globally, studies focusing on students have identified a moderate to high prevalence of depression, anxiety, and stress among this population during non-pandemic situations. Escobar-Viera et al., 2018, revealed that high rates of passive social media use were significantly related to higher depression, whereas more active use was associated with lower levels of depression [23]. Similarly, Drouin et al., 2018, opined that individuals with high levels of anxiety or depression cited the use of social media as a source of stress [24]. That notwithstanding, as the use of social media continues to be an effective channel for communicating and networking with friends and families [25,26,27], people with stress or anxiety are likely to use social media at even greater rates. This is particularly true, especially at a time where lockdown restrictions prevent people from having face-to-face social interactions.

Despite the existence of a handful of these studies into the effect of social media use on users’ mental health, none of those studies have focused on the impact of social media use on the everyday emotions of international students in the COVID-19 pandemic. Thus, there is the need to examine the impact social media use has on the everyday emotion (anxiety and depression) of international students in COVID-19.

## 2. Theoretical Background

Anxiety is a physiological state consisting of cognitive, somatic, emotional, and behavioral components [28]. The combination of these states creates the feeling of fear, apprehension, or worry. Anxiety is usually followed by physical sensations such as nausea, heart palpitations, chest pain, shortness of breath, headache, and stomach aches. Recent studies have found anxiety and depression disorders to be the second leading cause of disability among psychiatric disorders, especially in adults [29]. This is especially true during the lockdown situations of COVID-19, where the various avenues for reducing stressors that cause anxiety and depression become limited or virtually non-existent, making social media the most available source of reducing stress.

Research into the impact of social media use on users has produced mixed results over the years. On the one hand, social media use has been found to increase self-esteem, improve general well-being, enhance social support, reducing anxiety and depression stressors, and provide overall life satisfaction [30,31,32,33,34,35,36]. Other contrary studies have found that concerns of addiction, spreading of negative emotions, rumors, misinformation, and fake news on social media, turn to act as significant stressors rather than “anti-stressors” for anxiety and depression [21,22]. Hence, there is the need to interrogate further the role social media use plays in reducing or increasing the anxiety and depression of users in health crises, such as in the case of COVID-19.

Over the years, several psychotherapy tools have been introduced to assess and treat generalized anxiety disorder (GAD) and depression among individuals. One of such tools is cognitive-behavioral therapy (CBT). Cognitive-behavioral therapy has over the years become the “go-to” tool for treating several conditions and disorders, including antisocial behaviors (such as lying and stealing), anxiety disorders, depression, eating disorders (such as binge eating, anorexia, and bulimia), and general stress. Several studies have also documented CBT use in treating sleep disorders such as insomnia, excessive daytime sleepiness, etc. [37,38,39]. CBT is used to alter thought patterns to change these behaviors and moods [40]. The CBT tool is based on the rationale that an individual’s negative feelings or actions arise from current distorted thoughts or beliefs, not necessarily emanating from one’s unconscious forces from the past [40]. Experts use the CBT tool for both cognitive and behavioral therapy. The cognitive domain primarily focuses on ones’ moods and thoughts, whereas the behavioral domain is centered on an individual’s behaviors and actions.

As previous studies have found the use of social media during COVID-19 to impact everyday emotion (anxiety and depression), this study categorizes these emotions into the physiological, cognitive, behavioral, and emotional domains, which are consistent with the CBT model. The decision to use the CBT model stems from the fact that it is combinatory. It combines cognitive and behavioral therapies and has strong empirical evidence for treating mood and anxiety disorders [41,42,43].

Though the CBT model is a treatment tool, it is essential to identify and categorize everyday emotions relating to social media use into the CBT categories. This will help determine whether or not social media use impacts the various domains of CBT, i.e., physiological, behavioral, cognitive, and emotional domains, differently. Therefore, this study uses CBT as an identification tool rather than a therapeutic tool.

Four key domains interact within the human environment: the physiological domain made up of one’s biology, genetics, physical, and physiological attributes, and the behavioral domain characterized by an individual’s action and behavior. The cognitive domain consists of thoughts, cognitions, beliefs, and self-talk, while the emotional domain is made up of one’s feelings, moods, and emotions. Because an individual’s thoughts, feelings, actions, and physiological characteristics are so closely intertwined, any effects or changes in any of these domains impact others. Hence, adopting a more holistic approach to examining the impact social media use has on all these domains and, consequently, users’ everyday emotion is essential [37].

An individual’s anxiety and depression are triggered by several factors acting as stressors. Studies have found demographic factors such as age and sex to correlate with social media use [44]. Previous studies have shown a moderating role of age on the relationship between social media use and everyday emotion [45,46,47]. According to Hardy et al., 2018, social media tends to help young adults to cope with stressors; thus, improving their mental health, as it provides the channel for them to actively contribute content and avoid social isolation, which is not the case with those in their mid-30′s and 40′s [44]. Therefore, it is essential to evaluate whether age can predict the everyday emotion of international students who use social media.

Furthermore, sex has also been found to be crucial in the study of social media use and everyday emotion. For instance, studies have found a high combined use of social media and information technology to be associated with an increased risk of prolonged stress and symptoms of depression among women [48,49]. Other researchers have also found sex to moderate the relationships between stress and social support to physical health in college students [50,51]. Seeing that one’s sex is crucial to how they use and inadvertently handle anxiety and depression emanating from social media use, it is imperative to assess the role sex plays in the mix between social media use (SMU) and everyday emotion (EE) in COVID-19. Thus, to achieve the objective of this study, the following hypotheses were posited:

**Hypothesis** **1** **(H1).** *International students’ social media use has a significantly positive effect on their everyday emotions in COVID-19.*

**Hypothesis** **2a** **(H2a).** *Age has a significantly positive effect on international students’ everyday emotions in COVID-19.*

**Hypothesis** **2b** **(H2b).** *Age significantly moderates the relationship between international students’ social media use and their everyday emotion in COVID-19, such that the higher the age of a user, the weaker the relationship.*

**Hypothesis** **3a** **(H3a).** *Sex (Male/Female) has a significant effect on international students’ everyday emotions in COVID-19*.

**Hypothesis** **3b** **(H3b).** *Sex significantly moderates the relationship between international students’ social media use and their everyday emotion in COVID-19, such that the differences in the category of sex weaken the relationship between social media use and everyday emotions.*

**Figure 1:**
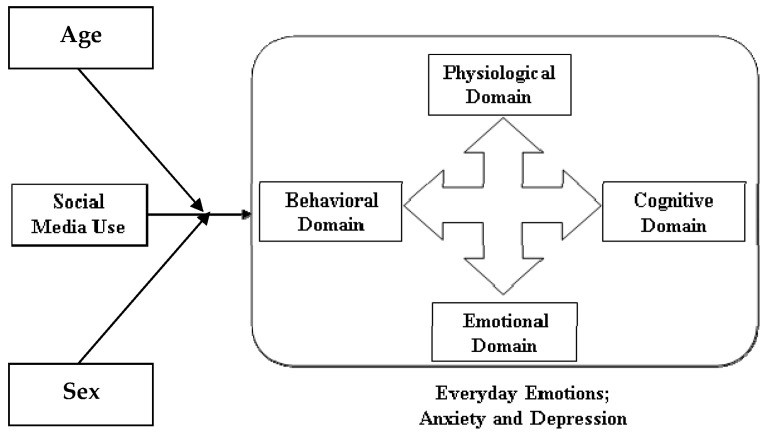
Model of SMU-EE.

The model in Figure 1 below is a representation of what this study seeks to explore.

## 3. Methods and Methodology

### 3.1. Instrument Design and Distribution

A structured online questionnaire consisting of questions adapted and modified from the General Anxiety Disorder-7 (GAD-7) instrument [52,53] and Patient Health Questionnaire (PHQ-9) [54], categorized into the four cognitive behavior therapy domains—physiological, behavioral, cognitive, and emotional domains—, together with social media use and demographic questions were used to collect data from respondents.

The questionnaire was developed in English using SurveyHero (http://www.surveyhero.com (accessed on 27 January 2020)), an online survey content management portal, setting restrictions such that respondents could answer the questions only once.

The questionnaire was divided into two sections; the first section contained an introduction stating the objective of the survey and the eligibility criteria for participating in the study, and the demographic information explicitly. The second portion included questions adapted from existing literature relating to the different constructs, as presented in the conceptual model in Figure 1, using a 5-point Likert scale. Table 1 below shows the questionnaire composition as it was adapted from literature sources (see Appendix A for actual questionnaire used to collect data for the study).

Respondents were informed about the research procedure, the voluntariness of their participation, privacy, and an option to opt-out of the survey at will at any point in time.

The questionnaire was distributed on various social media platforms; WeChat, Facebook, WhatsApp, and other relevant social media platforms.

### 3.2. Methods

#### 3.2.1. Sample

The respondents were international students who were studying in universities and colleges in various cities of China. The data were collected from January 2020 to May 2020.

A priori sample size calculator for structural equation model was used to compute the sample size required [56] for the study. The number of observed and latent variables in the model was set to 4 and 10, the anticipated effect size and the desired probability set at 0.3 and 0.05, respectively, using statistical power levels of 0.8. The calculator returned 137 as the minimum sample size required to detect the specified effect and 288 as the minimum sample size required given the structural complexity of the model.

Of the total 480 responses received, six were excluded due to either substantially incomplete responses or missing values. A total of 474 responses were used for further analysis. For statistical analysis such as structural equation modeling (SEM), a sample size of 200 is considered to be fair and 300 as good [57,58,59,60]. Hence, the sample size of 474 meets these requirements and was significant enough to obtain robust results after analysis.

#### 3.2.2. Data Analysis

Data from questionnaires were exported from the online survey content management portal into Microsoft excel for cleaning and imported into IBM SPSS Statistics version 23 and Intellectus Statistics online software for statistical analysis. The data analysis included descriptive and partial least squares path modeling (PLS-PM model) analysis.

Cronbach’s alpha was used to test the reliability of the various constructs using the guidelines suggested by George and Mallery, 2018, where >0.9 is excellent, >0.8 is good, >0.7 is acceptable, >0.6 is questionable, >0.5 is poor, and ≤0.5 is unacceptable [61].

The PLS-PM model was assessed by evaluating the validity of the measurement model and the structural model. After model validation, the regressions of the PLS-PM were analyzed.

The measurement model (outer model) was assessed by examining the unidimensionality, loadings, communalities, and cross-loadings of the indicator variables. Bootstrapping was also used to check the significance of each loading.

Unidimensionality of Indicators. For reflective indicators, the latent construct must be positively correlated with each indicator. If the latent variable increases in value, then each indicator should also increase. Sanchez (2013) defines this as the unidimensionality of indicators [62]. Cronbach’s alpha (α) and Dillon-Goldstein’s rho (ρ) were calculated to evaluate the unidimensionality of indicators. Unidimensionality of indicators could be assumed if Cronbach’s alpha and Dillon-Goldstein’s rho had large values (α ≥ 0.7 and ρ ≥ 0.7).

Factor Loadings and Communality. The factor loadings and communalities were examined for the reflective indicators to identify indicators with weak loadings for the latent variables. The variability in each indicator should explain at least 50% of its latent variable construct (|loading| ≥ 0.707; communality ≥ 0.50) [62,63,64]. Otherwise, it is identified as a weak loading.

Cross-Loadings. The cross-loadings were also examined for the reflective indicators to assess the validity of the model. A cross-loading occurs when an indicator has a higher absolute loading on a different latent variable than the one to which it is assigned [62,64,65].

Bootstrapping. Bootstrapping was performed with 500 resamples. The loadings were assessed for the reflective indicators, and the weights were examined for formative indicators. Significance was determined using 95% confidence intervals for the given parameter estimates, which were calculated based on an alpha value of 0.05 [62,63,64].

The structural or inner model was assessed by examining the R^2^-values for each endogenous variable, the average variance extracted (AVE) for each latent variable with reflective indicators, and the goodness of fit (GoF) index for the model. Bootstrapping was also used to determine the reliability of the inner model.

R^2^-values. The R^2^-values were calculated for each endogenous variable to determine if the relationships among the latent variables were appropriate. Each endogenous variable should have an R^2^-values ≥ 0.20 [62].

Average Variance Extracted. The average variance extracted for each construct was calculated to verify whether each latent variable had a strong relationship with its reflective indicators. Each latent variable should have an AVE ≥ 0.50, which suggests that 50% or more of the variance for the indicators is explained by its latent variable [62,63,64]. AVE is only assessed for reflective variables.

Goodness of Fit. The GoF index was used to assess the predictive power of the PLS-PM. The GoF index was calculated by computing the geometric mean of the average R^2^-values and average commonality for each latent variable. Values greater than 0.90 are considered a good model fit, while a GoF index less than 0.90 and greater than 0.70 is an acceptable model fit [62,63]. A model with poor predictive power is indicated by a GoF index less than or equal to 0.70.

Bootstrapped Regression Paths. Bootstrapping was performed with 500 resamples. The regression coefficients were evaluated using 95% confidence intervals to determine the significance of the regression paths using an alpha value of 0.05 [62,63,64].

Bootstrapped Moderating Effects. Bootstrapping was performed with 500 resamples. Moderation was evaluated using the two-stage approach to create each interaction term from the latent variable scores [62]. The moderating effects were assessed using the significance of each interaction term with 95% confidence intervals.

## 4. Results

### 4.1. Sample Demographic Characteristics

The demographic distribution of the respondents is presented in Table 2. The most frequently observed age category was 24 to 30 (*n* = 154, 32.50%). The majority of the respondents were pursuing their bachelor’s degree (*n* = 229, 48.30%) at the time of this study. The majority of respondents were residents in Zhenjiang (*n* = 209, 44.10%), while the most frequently observed category of sex was Male (*n* = 278, 58.60%). Furthermore, the most frequently observed category for the number of years resident in China was two years (*n* = 182, 38.40%). Finally, the result showed that the most frequently observed category for nationality was Ghana, with a total number of respondents (*n* = 94, 19.80%).

### 4.2. Reported Anxiety and Depression Levels

The overall anxiety and depression ratings of social media users in COVID-19 as assessed using the GAD-7 and PHQ-9 measuring instruments are presented in Table 3. The most reported anxiety level was MILD (*n* = 249, 52.50%), while the most reported level of depression was also MILD (*n* = 350, 73.80%).

### 4.3. Reliability Test for the Scales Used to Assess the Impact of Social Media on Everyday Emotions

A Cronbach alpha coefficient was calculated for the various scales used to assess the impact of social media on everyday emotion, consisting of GAD-7, PHQ-9, and social media use.

The items for the GAD-7, PHQ-9, and social media use scales had Cronbach’s alpha coefficients of 0.85, 0.76, and 0.79, respectively, indicating an excellent internal reliability compared to other studies [54,66]. Table 4 presents the results of the reliability analysis and the item-total statistics.

### 4.4. Partial Least Squares Path Modeling

**Figure 2:**
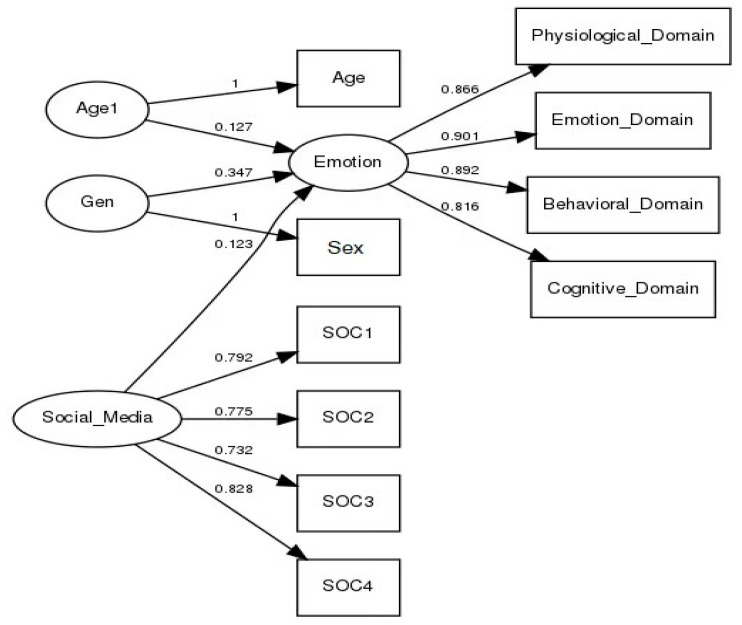
Node diagram for the SMU-EE model with loadings shown.

A partial least squares path modeling (PLS-PM) analysis was conducted to determine whether the latent variables, social media use, everyday emotion, sex, and age, adequately described the data. The goal of PLS-PM is to describe the network of variables and their relationships accurately. The PLS-PM model diagram can be seen in Figure 2.

#### 4.4.1. Measurement Model Summary

Unidimensionality of Indicators. All latent variables exhibited unidimensionality, indicating the relationships between the latent variables and indicators were appropriate for PLS-PM. The unidimensionality assumption did not apply to latent variables with only one indicator variable. The Cronbach’s alpha and Dillon-Goldstein’s rho statistics are presented in Table 5.

Factor Loadings and Communality. Table 6 presents the loadings and communalities for the measurement model. There were no reflective indicators with weak loadings, indicating that each reflective indicator explained a significant portion of the variance in its latent construct.

Cross-Loadings. Table 7 shows results obtained for cross-loadings. There were no cross-loadings for reflective indicators in the model, suggesting the specified latent variable structure was appropriate for the data.

Bootstrapping. Each reflective manifest variable had a significant loading, suggesting that its latent variable explains a substantial portion of each reflective indicator. Since there were no formative indicators, the bootstrapped weights were not examined. Table 8 shows the results for the bootstrapped loadings.

#### 4.4.2. Summary of the Structural Model

**Figure 3:**
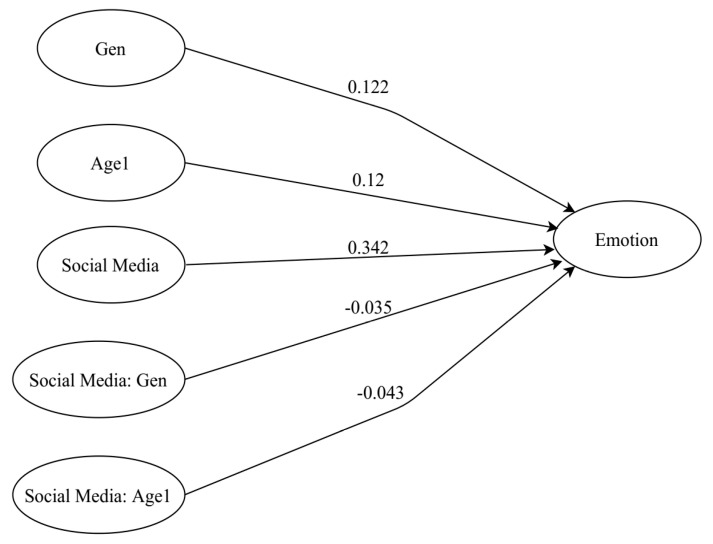
Inner node diagram for the SMU-EE model.

The table of the inner model summary is presented in Table 9, and the inner model node diagram is shown in Figure 3.

R^2^-values. Table 9 shows the R^2^-values for the endogenous latent variable; emotion was 0.14, which is considered a weak explanatory power [62].

Average Variance Extracted. As presented in Table 9, results of AVE values show that there were no latent variables with a low AVE, indicating that each latent variable accounted for a significant portion of the indicator’s variance.

Goodness of Fit. The GoF index, GoF = 0.31, indicates that the model had a poor model fit and poor prediction ability.

Bootstrapped Regression Paths. Social media use significantly predicted everyday emotion, B = 0.34, 95% CI (0.26, 0.44). Age was also found to significantly predict everyday emotion, B = 0.12, 95% CI (0.03, 0.21). Additionally, there was a statistically significantly relationship between the category of sex and everyday emotion (B = 0.12, 95% CI (0.04, 0.21)). Thus, hypotheses H1, H2a, and H3a were supported. Table 10 shows the regression results for the inner model with bootstrapping.

Bootstrapped Moderating Effects. Regarding the moderating effects, this study found that sex and age did not significantly moderate the effect social media use had on everyday emotion (B = −0.03, 95% CI (−0.12, 0.06), B = −0.04, 95% CI (−0.15, 0.08)), respectively; thus, not satisfying H2b and H3b. Table 10 shows the inner model results, which include any moderating effects.

## 5. Discussion

This study aimed to evaluate the impact of social media use on everyday emotion (anxiety and depression) of international students during the outbreak of the COVID-19 pandemic. The study found a significant positive impact of social media use on everyday emotions of international students, as shown in Table 10. This finding was consistent with other studies that have examined the effect of social media use on anxiety and depression [11,67,68].

Studies on the effect of social media use on anxiety and depression have had, over the years, reported mixed results. While some studies have shown that social media provides benefits such as enabling individuals to express their thoughts and feelings and receive social support [26,69,70], others have also demonstrated a relationship between social media use and health problems [15,16,17].

At a critical time such as this, when quarantining and social distancing are becoming the norm in several parts of the world [17,71], social media has become a valuable tool for harnessing information to increase the mobilization drive of communities to follow quarantine procedures. Social media has helped decrease fears and uncertainty and enhance public trust in public health measures. According to Ni et al., 2020, social media use in COVID-19 can be a tool to overcome the social distancing constraints during quarantine and provide mental health support resources and solidarity with those persons in a lockdown situation [14,72]. Social media offers a platform for international students to consistently stay in touch with their families and friends and other social interactions as a means of building social support systems and alleviate depression and anxiety [73].

The use of social media among international students may have presented students with the opportunity to provide reassurance and practical advice within their social networks to pre-empt panic and rumors [72]; hence, improving their anxiety and depression significantly. Studies have shown that social media use can facilitate forming connections among marginalized and stigmatized individuals because of health conditions such as anxiety and depression [73].

Conversations on social media present a unique opportunity to promote the awareness of COVID-19, its symptoms, and the possible effects of institutional decisions such as quarantine measures and the development of COVID-19 vaccines. This information keeps international students informed; thus, reducing the stressors and fears caused by COVID-19. Studies on information use and social media use have shown that a lack of information in crucial times could increase tension, causing anxiety and depression [74]. Additionally, the downward trend in the overall epidemic curve of COVID-19 in China suggests the broadcast of critical information on social media, aimed at promoting hand washing, mask-wearing, and care-seeking with high frequency through multiple channels [75] may have significantly contributed to the overall effect of social media use among international students.

This study found a significantly positive correlation between all the constructs; behavioral domain, physiological domain, cognitive domain, and emotional domain of international students. These findings are consistent with previous studies [31,76,77] that have examined the relationship between these constructs.

Sex and age were introduced into the model to test their moderating effect on the relationship between social media use and everyday emotion (anxiety and depression) during the COVID-19 pandemic. The two categories of sex and the ages of social media users did not significantly moderate the relationship between social media use and everyday emotions, contrary to other studies [50,51]. It is possible that, because in the peak of the pandemic, social media users across the categories of sex and ages had access to a similar form of information on their social media platforms. Furthermore, it appeared that users might have been thinking of the same issues, i.e., COVID-19-related information and issues; thus, the overall effects of sex and age on the everyday emotions of international students were not significant.

Finally, actual reported levels of anxiety and depression associated with social media use among international students in COVID-19 were generally not severe. Users reported predominantly mild levels of stress assessed with GAD-7 and depression assessed with PHQ-9, as shown in Table 3.

## 6. Conclusions

This study found social media use during the COVID-19 pandemic to positively impact international students’ everyday emotions. This finding contradicts other studies that have found excessive use of social media to negatively impact users’ behaviors, especially among young people and “mobile-borns.” The study emphasized the immerse use of social media among international students to curb the adverse effect of everyday emotions, which seems to be a significant mental health threat among students. Based on the findings of this current study, it is suggested that stakeholders continue to implement programs such as online mental health consultations, counseling programs, and public health education campaigns for maintaining social support and enhancing public trust in public health measures. The continuation of these programs will help consolidate the gains made in pre-empting and eliminating anxiety and depression associated with social media use among international students. 

Furthermore, it is also imperative for international students to avail themselves to access these existing programs and other ones that stakeholders roll out in the future to safeguard their holistic well-being in COVID-19. Finally, to the best of our knowledge, this is the first study on international students’ everyday emotions and social media use in the era of the COVID-19 pandemic. We also believe that these results are highly representative of, and generalizable to, the population of international students in China, owing to the standardized procedures of data collection and findings established.

## 7. Limitations and Future Study

This study had three main limitations which could be addressed in future research. First, the study focused solely on the impact of social media use on international students’ everyday emotions during the pandemic. However, understanding the influence of social media use on everyday emotion in non-pandemic situations will be extremely useful. Such studies will help determine whether there are differences in the magnitude of the impact of social media use on everyday emotion in the two situations. Second, this study could not ascertain whether or not the relationship between social media use and everyday life among international students and Chinese students is similar. Future research should compare a sample of international students with a similar sample of Chinese students to see whether there are any parallels in the influence of social media use on everyday emotion between these two groups. Lastly, “mobile-borns” are perceived to be at a high risk of addiction due to their excessive exposure to social media in the pre-pandemic period. This exposure may have enabled them to develop defense mechanisms to cope with social media-related anxiety and depression in the pandemic. However, this current study did not explore this dynamic. Thus, future studies may consider exploring this dynamic further.

## Figures and Tables

**Table 1:** Measurement instruments.

Constructs	# of GAD-7 Questions	Notations		# of PHQ-9 Questions	Notations		Literature Sources
Physiological Domain (PHY)	2	PHY1-PHY2		1	PHY3		[53,54]
Emotional Domain (EM)	3	EM1-EM3		5	EM4-EM8		[53,54]
Behavioral Domain (BEH)	1	BEH1		-	-		[53,54]
Cognitive Domain (COG)	1	COG1		3	COG2-COG4		[53,54]
**Social Media Use Construct**							
**Construct**	**# of Questions**		**Notations**			**Literature Sources**	
Social Media Use (SOC)	4		SOC1-SOC4			[55]	

**Table 2:** Socio-demographic characteristics of respondents.

Variable	*N*	%		
**Age**			**Median**	**Std. Deviation**
18 to 23	167	35.20	24	6.21
24 to 30	154	32.50		
31 to 36	112	23.60		
37 to 42	41	8.61		
**Sex**				
Female	196	41.40		
Male	278	58.60		
**Marital Status**				
Married	111	23.40		
Single	355	74.90		
Divorced	8	1.70		
**Level of Education**				
Diploma	16	3.40		
Bachelor’s degree	229	48.30		
Master’s degree	155	32.70		
Doctorate	74	15.60		
**City of Residence in China**				
Beijing	25	5.30		
Nanjing	55	11.60		
Shanghai	53	11.20		
Suzhou	31	6.50		
Wuhan	35	7.40		
Zhenjiang	209	44.10		
Others *	66	13.90		
**Years Resident in China**			**Median**	**Std. Deviation**
1 year and below	41	8.60	3	1.26
2 year	182	38.40		
3 years	96	20.30		
4 years	73	15.40		
5 years and above	82	17.30		
**Nationality**				
Bangladesh	33	7.00		
Cameroon	30	6.30		
Cote D’Ivoire	26	5.50		
Ethiopia	24	5.10		
Ghana	94	19.80		
India	70	14.80		
Jamaica	25	5.30		
Nigeria	42	8.90		
Pakistan	41	8.60		
United States of America (USA)	35	7.40		
Zimbabwe	29	6.10		
Others **	25	5.30		

**Note.** Due to rounding errors, percentages may not equal 100%; others *: Cities with less than 20 respondents (i.e., Chengdu, Guangzhou, Hangzhou, Shenzhen, and Yangzhou); others **: Countries with less than 20 respondents (i.e., Germany and Italy).

**Table 3:** Self-reported anxiety and depression levels of social media users in COVID-19.

**GAD-7 Scale**	***n***	**Percentage**
Very Mild	133	28.10
Mild	249	52.50
Moderate	88	18.60
Moderately Severe	4	0.80
**PHQ-9 Scale**	***n***	**Percentage**
Very Mild	13	2.70
Mild	350	73.80
Moderate	105	22.20
Moderately Severe	6	1.30

**Table 4:** Reliability table for everyday emotions scale and social media use items.

Scale				No. of Items		α		α Based on Standardized Items	
GAD-7				7		0.85		0.85	
PHQ-9				9		0.76		0.79	
Social Media Use				4		0.79		0.79	
	**Items**	**Scale Mean if Item Deleted**	**Scale Variance if Item Deleted**		**Corrected Item-Total** **Correlation**		**Squared Multiple Correlation**		**α if Item Deleted**
GAD-7	PHY1	10.51	17.78		0.52		0.33		0.84
	PHY2	9.96	16.23		0.59		0.42		0.83
	BEH1	10.30	16.24		0.63		0.46		0.83
	COG1	10.55	17.36		0.57		0.42		0.84
	EM1	10.23	16.21		0.65		0.45		0.83
	EM2	10.08	15.91		0.65		0.48		0.82
	EM3	10.28	16.19		0.67		0.48		0.82
PHQ-9	EM4	16.01	20.20		0.65		0.49		0.72
	EM5	15.73	20.14		0.49		0.30		0.74
	EM6	15.55	19.25		0.62		0.44		0.71
	EM7	15.66	19.74		0.63		0.44		0.71
	EM8	15.96	21.15		0.41		0.23		0.75
	COG2	15.67	19.25		0.68		0.52		0.71
	COG3	16.10	20.61		0.64		0.51		0.72
	COG4	15.81	20.89		0.46		0.25		0.74
	PHY3	13.86	27.31		−0.24		0.09		0.85
SMU	SOC1	6.35	7.32		0.59		0.34		0.75
	SOC2	6.39	7.33		0.62		0.39		0.73
	SOC3	6.27	6.98		0.58		0.34		0.75
	SOC4	6.38	7.04		0.62		0.38		0.73

**Table 5:** Unidimensionality of indicators for each latent construct.

Construct	Indicator Type	Number of Items	*α*	*ρ*
Social Media Use	Reflective	4	0.79	0.86
Everyday Emotion	Reflective	4	0.89	0.93
Sex	Reflective	1	--	--
Age	Reflective	1	--	--

Note: unidimensionality does not apply to formative indicators or latent variables with only one indicator variable.

**Table 6:** Outer model summary table for the PLS-PM model.

Indicator	Construct	Weight	Loading	Communality
SOC1	Social Media Use	0.35	0.79	0.63
SOC2		0.28	0.77	0.60
SOC3		0.25	0.73	0.54
SOC4		0.39	0.83	0.69
Physiological Domain	Everyday Emotion	0.25	0.87	0.75
Emotional Domain		0.29	0.90	0.81
Behavioral Domain		0.29	0.89	0.80
Cognitive Domain		0.33	0.82	0.67
Gen	Sex	1.00	1.00	1.00
Age1	Age	1.00	1.00	1.00

**Table 7:** Loadings and cross-loadings for the outer model.

Indicator	Social Media Use	Everyday Emotion	Sex	Age
SOC1	**0.79**	0.29	0.03	0.01
SOC2	**0.77**	0.23	−0.01	−0.07
SOC3	**0.73**	0.21	0.05	−0.11
SOC4	**0.83**	0.32	−0.00	−0.03
Physiological Domain	0.35	**0.87**	0.07	−0.02
Emotional Domain	0.27	**0.90**	0.10	0.10
Behavioral Domain	0.32	**0.89**	0.09	0.03
Cognitive Domain	0.26	**0.82**	0.11	0.14
Sex	0.02	0.11	**1.00**	−0.21
Age	−0.05	0.08	−0.21	**1.00**

Note: the bolded items are the specified loadings for each indicator.

**Table 8:** Bootstrap results for the loadings of each indicator.

Path	Original	M	SE	95% CI
Social Media Use → SOC1	0.79	0.79	0.03	(0.73, 0.85)
Social Media Use → SOC2	0.77	0.77	0.03	(0.70, 0.83)
Social Media Use → SOC3	0.73	0.73	0.04	(0.65, 0.80)
Social Media Use → SOC4	0.83	0.83	0.02	(0.78, 0.87)
Everyday Emotion → Physiological Domain	0.87	0.87	0.02	(0.84, 0.91)
Everyday Emotion → Emotional Domain	0.90	0.90	0.01	(0.88, 0.92)
Everyday Emotion → Behavioral Domain	0.89	0.89	0.01	(0.86, 0.92)
Everyday Emotion → Cognitive Domain	0.82	0.81	0.03	(0.73, 0.85)
Gen → Sex	1.00	1.00	0.00	(1.00, 1.00)
Age1 → Age	1.00	1.00	0.00	(1.00, 1.00)

Note: estimates based on 500 samples.

**Table 9:** Structural model summary.

Construct	Type	*R* ^2^	AVE
Social Media Use	Exogenous	--	0.61
Everyday Emotion	Endogenous	0.14	0.76
Sex	Exogenous	--	1.00
Age	Exogenous	--	1.00

Note: for constructs with formative factors, AVE was not assessed; *R*^2^ was not calculated for exogenous variables.

**Table 10:** Bootstrap results for the inner model regression paths.

Path	Original B	M	SE	95% CI
Sex → Everyday Emotion	0.12	0.12	0.04	(0.04, 0.21)
Age → Everyday Emotion	0.12	0.12	0.04	(0.03, 0.21)
Social Media Use → Everyday Emotion	0.34	0.34	0.05	(0.26, 0.44)
Social Media Use: Gen → Everyday Emotion	−0.03	−0.03	0.05	(−0.12, 0.06)
Social Media Use: Age1 → Everyday Emotion	−0.04	−0.04	0.06	(−0.15, 0.08)

Note: ‘:’ indicates an interaction term; estimates based on 500 samples.

## Data Availability

Data for this study are available on request from the corresponding author and or first author.

## Appendix A. Questionnaire

**Modified GAD-7**					
	Never	Rarely	Sometimes	Frequently	Always
Do you feel nervous, anxious, or on edge whenever you receive a rumor or update of situations regarding the outbreak of COVID-19 on your social media platforms?					
	No	Yes, but not often	Yes, sometimes	Yes, frequently	Yes, all the time
Do you worry or are often unable to stop or control your worrying whenever you receive an alert, a rumor, or an update of situations regarding the outbreak of COVID-19 on your social media platforms?					
	No, never	Yes, but rarely	Yes, sometimes	Yes, often	Yes, all the time
Do you worry too much about different things whenever you see an update of situations regarding the outbreak of COVID-19 on your social media platforms?					
	No, never	Yes, but rarely	Yes, sometimes	Yes, often	Yes, all the time
Do you have trouble relaxing whenever you are on social media and you see an update of situations regarding the outbreak of COVID-19?					
	No, never	Yes, but not often	Yes, sometimes	Yes, often	Yes, always
When you are on a particular social media platform where there’s no update on COVID-19, do you feel so restless that it becomes hard for you to sit still or find yourself leaving that platform to other ones to see if there are updates regarding COVID-19, just in case?					
	No, never	Yes, but rarely	Yes, sometimes	Yes, often	Yes, all the time
Do you become easily annoyed or irritated after receiving updates of situations regarding the outbreak of COVID-19 on social media?					
	No, never	Yes, but not often	Yes, sometimes	Yes, often	Yes, always
Do you feel afraid as if something awful might happen whenever you are on your social media platform since the outbreak of COVID-19?					
	Never	Rarely	Sometimes	Often	All the time
How often are you nervous when using social media since the outbreak of COVID-19?					
**Modified PHQ-9**					
	No, never	Yes, but rarely	Yes, sometimes	Yes, often	Yes, all the time
Do you lose interest or lose pleasure in doing general chores or things whenever you receive updates regarding the outbreak of COVID-19 on your social media platforms?					
	No, never	Yes, but rarely	Yes, sometimes	Yes, often	Yes, all the time
Do you feel down, depressed or hopeless when someone posts any new articles or rumors concerning COVID-19 on a social media platform you are on?					
	No, never	Yes, but rarely	Yes, sometimes	Yes, often	Yes, all the time
Do you have trouble falling asleep, staying asleep, or sleeping too much after someone posts new articles or rumors concerning COVID-19 on a social media platform you are on?					
	Never	Rarely	Sometimes	Often	All the time
Do you feel tired or having little energy or overwhelmed after reading new articles or rumors concerning COVID-19 on a social media platform you are on?					
	No, never	Yes, once in a while	Yes, sometimes	Yes, often	Yes, all the time
Do you have a poor appetite or find yourself overeating after reading new articles or rumors concerning COVID-19 on a social media platform you are on?					
	No, never	Yes, but not often	Yes, sometimes	Yes, often	Yes, always
Have you been feeling bad about yourself or that you’re a failure or have let yourself or your family down after reading new articles or rumors concerning COVID-19 on a social media platform you are on?					
	No, never	Yes, but not often	Yes, sometimes	Yes, often	Yes, always
Do you have trouble concentrating on things, such as reading the newspaper or watching television after reading new articles or rumors concerning COVID-19 on a social media platform you are on?					
	No, never	Yes, but not often	Yes, sometimes	Yes, often	Yes, always
Do you find yourself moving or speaking so slowly that other people could have noticed? Or, do you find yourself being so fidgety or restless that you have been moving around a lot more than usual when you read new articles or rumors concerning COVID-19 on a social media platform you are on?					
	Never	Rarely	Sometimes	Often	Always
Do thoughts that you would be better off dead or of hurting yourself in some way come into your cognition when you read new articles or rumors concerning COVID-19 on a social media platform you are on?					
**Social Media Use**					
	Strongly disagree	Disagree	Neither agree nor disagree	Agree	Strongly agree
I keep reading/watching social media content relating to COVID-19 to forget about personal problems					
	Strongly disagree	Disagree	Neither agree nor disagree	Agree	Strongly agree
I have tried to limit my usage of social media to search for content relating to COVID-19 without success					
	Strongly disagree	Disagree	Neither agree nor disagree	Agree	Strongly agree
I become restless or troubled if I am prohibited from using social media to search for health information on COVID-19					
	Strongly disagree	Disagree	Neither agree nor disagree	Agree	Strongly agree
I have used social media to search for health information relating to COVID-19 so much that it has had a negative impact on my life.					
**Miscellaneous**					
	No	Yes, but only three times	Yes, a few more times than twice	Yes, many more times than twice	Yes, so many times I’ve lost count
Have you been to your doctor or the emergency department at the hospital or spoken to a friend about how anxious you are whenever you receive a rumor or update of situations regarding the outbreak of COVID-19 on your social media platforms more than twice?					
	Not useful	Less useful	Neither useful nor useless	Useful	Very useful
How would you rate the usefulness of social media platforms in epidemic situations					
